# Investigation with able-bodied subjects suggests Myosuit may potentially serve as a stair ascent training robot

**DOI:** 10.1038/s41598-023-35769-2

**Published:** 2023-08-29

**Authors:** Jaewook Kim, Yekwang Kim, Seonghyun Kang, Seung-Jong Kim

**Affiliations:** Department of Biomedical Engineering, Korea University College of Medicine, Seoul, 02841 Korea

**Keywords:** Biomedical engineering, Rehabilitation

## Abstract

Real world settings are seldomly just composed of level surfaces and stairs are frequently encountered in daily life. Unfortunately, ~ 90% of the elderly population use some sort of compensation pattern in order to negotiate stairs. Because the biomechanics required to successfully ascend stairs is significantly different from level walking, an independent training protocol is warranted. Here, we present as a preliminary investigation with 11 able-bodied subjects, prior to clinical trials, whether Myosuit could potentially serve as a stair ascent training robot. Myosuit is a soft wearable exosuit that was designed to assist the user via hip and knee extension during the early stance phase. We hypothesized that clinical studies could be carried out if the lower limb kinematics, sensory feedback via plantar force, and electromyography (EMG) patterns do not deviate from the user’s physiological stair ascent patterns while reducing hip and knee extensor demand. Our results suggest that Myosuit conserves the user’s physiological kinematic and plantar force patterns. Moreover, we observe approximately 20% and 30% decrease in gluteus maximus and vastus medialis EMG levels in the pull up phase, respectively. Collectively, Myosuit reduces the hip and knee extensor demand during stair ascent without any introduction of significant compensation patterns.

## Introduction

Over the years, it has been repeatedly emphasized that independent ambulation directly relates to quality of life^1–4^. Therefore, physical therapy for post-injury and elderly exercise has focused primarily on walking^5–8^. Unfortunately, real-world environments are not composed of just level surfaces. In order to reach “high-level of independence” and freely navigate one’s environment, the ability to not only perform overground level gait but also negotiate curbs and flights of stairs is imperative^9,10^.

To the untrained eye, walking up a flight of stairs and over a level surface may appear to be similar. However, from a bio-mechanical perspective the two are completely different. While the transfer between kinetic and potential energy during overground gait minimizes energy consumption, constant work against gravity must be performed in order to ascend a flight of stairs^11–13^. When closely examined, overground walking involves the transfer from ipsilateral heel to forefoot rocker and passive swinging of the contralateral limb^14,15^, while stair ascent relies on pushing of the ground with the extension of the ipsilateral hip and knee and active pulling of the contralateral limb^12,16,17^. Thus, different tasks are required to successfully complete each activity, which also implies that the gait phases that constitute a single walking or stair ascent period are different from each other^9,10^. This is also evident from the muscle recruitment patterns as stair ascent depends highly on knee and hip extensor muscles such as vastus medialis (VM), etc. while overground gait is mediated mainly by the tibial muscles^16,18^.

Unfortunately, because of its innate difficulty of stair ascent due to requiring much more physical aptitude such as range of motion (ROM), muscle strength, balance control, and cardiopulmonary function^10,18–22^, stair ascent training is not as heavily incorporated into the rehabilitation regimen as overground gait. The increased risk of falling and the severity of the injuries^9,23,24^, added difficulty for the physical therapist to prevent loss of balance on a flight of stairs, and psychological factors such vitality, anxiety, and fear of falling^25,26^ also limit stair training. Moreover, accurate depth perception as well as proprioception of the foot are required in order to clear and safely place the foot onto the next step^27,28^.

Currently, stair negotiation training not only focuses on strengthening the relevant muscles but also adheres to the basic motor learning principles which involves volitional repetition of task-specific movements provided with accurate feedback^29–31^. Considering that the loss of plantar sensitivity is independently associated with the risk of falling^32–34^, appropriate cues, sensory feedback as well as performance accuracy are vital when facilitating rehabilitative efficacy. These principles have been shown to optimize recovery under the premise that the weight-bearing task be performed by patient’s own legs with minimal assistance^35–37^. Traditional rehabilitation sessions rely heavily on physical therapists and weight bearing harnesses. This puts too much burden on the therapists and moreover the patient is unable to train as frequently due to limited resources^38^. Furthermore, a paradigm shift is warranted considering that over 90% of the elderly population use some sort of compensation pattern in order to negotiate stairs^39–42^. These statistics are indicative of severe social problems as super-aged society seems to be imminent.

Advancements in rehabilitation robots have brought some overdue excitement to stair negotiation rehabilitation. Traditional hard exoskeleton type robots and stationary end-effector types have started the movement. However, the lack of volitional movement and non-physiological sensory feedback of the foot have been identified to be responsible for the lackluster results^36,43,44^. Furthermore, the high inertia of the devices elicits unwanted non-physiological muscle activation patterns^43,45–47^. On a bright note, a recent study by Bannwart et al. demonstrated that a ceiling mounted robotic body weight support system could be potentially used as a stair ascent training device by showing that the task demand was reduced, evident by decreased EMG levels, while not affecting the user’s natural kinematics^9^. This adheres well to the hypothesis that training should be task related and that compensatory movements should not be introduced while lowering the task demand. However, theses devices require a large foot print, thus, may not be feasible in real-world situations^9^. An alternative solution to this problem could be a soft wearable robot that is portable, back-drivable with low inertia, and possibly be deployed in various environments.

**Figure 1:**
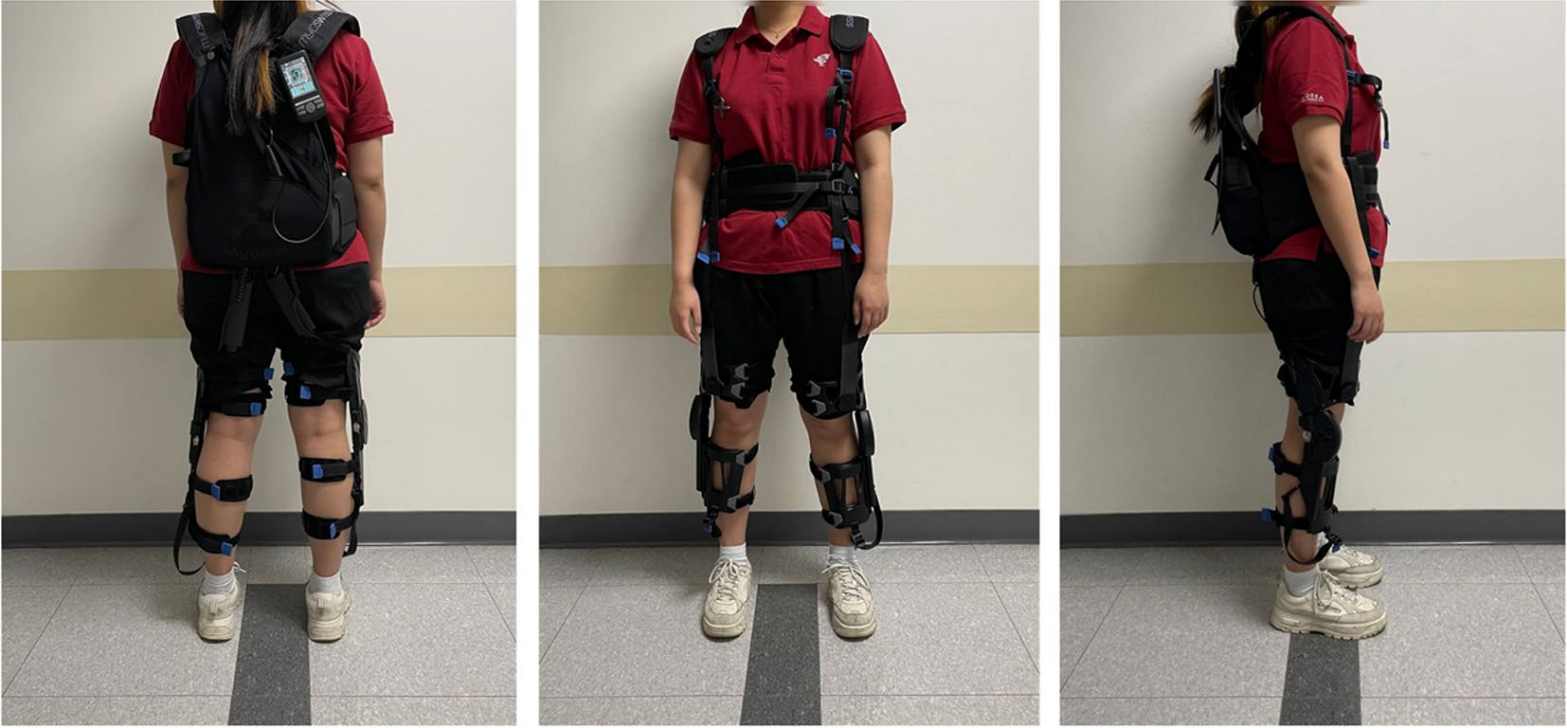
Subject wearing Myosuit. The posterior, anterior, and sagittal view of a subject wearing Myosuit.

**Figure 2:**
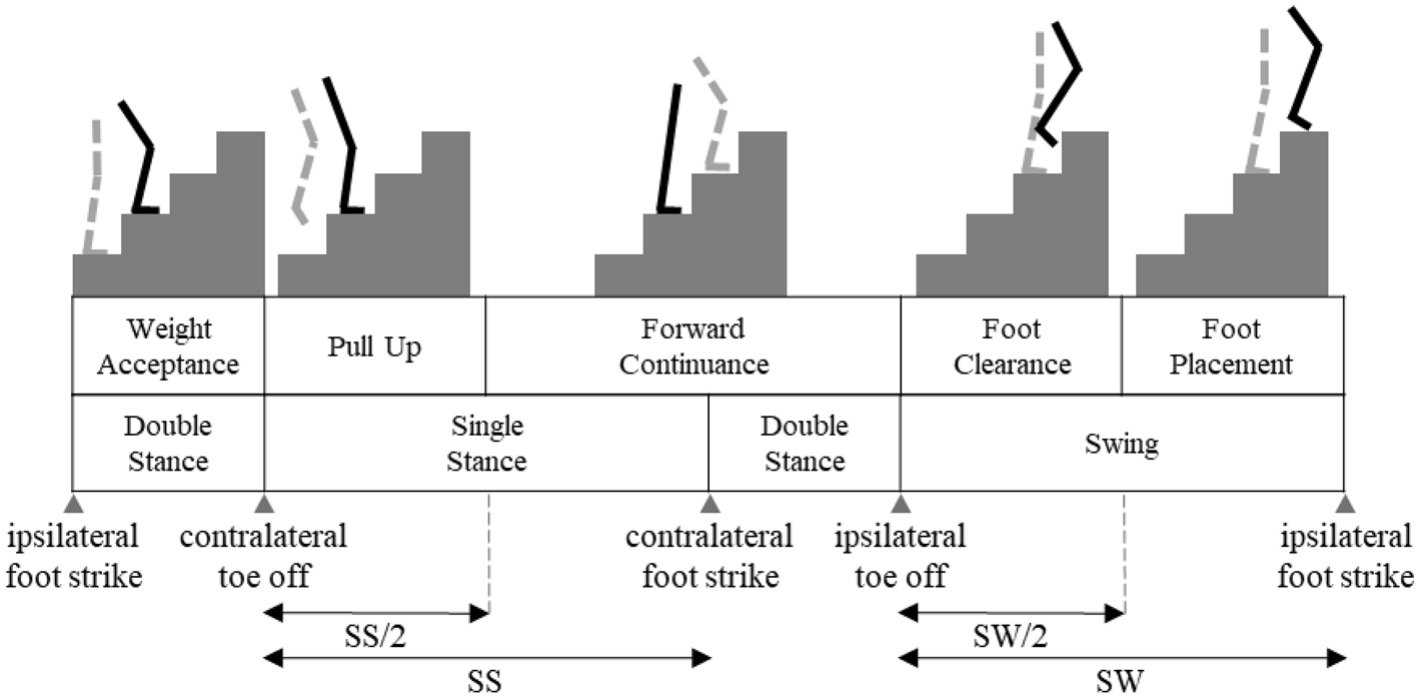
Stair ascent gait cycle. The 5 phases of the gait cycle during stair ascent are shown. The stance phase is further divided into 3 distinct phases: WA, PU, and CN phase. The ipsilateral foot-strike initiates the WA phase, the PU and CN phase is defined as half of the single leg support region (SS), and CN phase lasts until ipsilateral toe-off. The swing phase (SW) can be divided equally into FC and FP phase.

This paper aims to serve as a preliminary investigation of the changes elicited in the lower limb kinematics, plantar force patterns, and EMG activity of healthy subjects while walking up a flight of stairs and wearing Myosuit^48–50^ (Fig. 1). Our goal was to assess whether Myosuit causes any non-physiological kinematic or sensory patterns via plantar force and, furthermore, if the task demand was reduced. We hypothesize that if Myosuit does not induce large deviations that alter the individual’s lower limb kinematics, plantar force, and EMG patterns while lowering hip and/or knee extensor EMG levels, Myosuit might be a viable solution for stair ascent training. In order to evaluate the data according to phases involved with stair ascent, we dissected the gait cycle into five non-overlapping phases: weight acceptance (WA), pull up (PU), forward continuance (CN), foot clearance (FC), foot placement (FP)^12,51–55^ (Fig. 2). The results, from 11 healthy subjects, suggest that Myosuit well assists the user by lowering the hip and knee extensor demand during the WA and PU phase, as designed, without interfering with the user’s physiological patterns.

## Methods

### Myosuit

Myosuit, is a portable soft wearable robot that is approximately 5.6 kg in weight^48^ (Fig. 1). It was designed to function as an external muscle that assists the user with hip and knee extension during various activities of daily life. A backpack unit which is worn by the user contains two motors, a battery, and a controller. Two cables, one for each leg, extend out from the backpack unit and anchors at the distal thigh and proximal shank. Of the various control modes offered by Myosuit, “Transparency” and “Assist” mode was used in this study. We need to mention that the “Transparency” mode is not completely transparent to the user, it is a mode that controls the cables with minimal force but without any slack^49^. And with “Assist” mode, Myosuit identifies the gait phase using a built-in IMU sensor and operates such that knee and hip extension torque is actively applied during the early stance phase^48–50^. The amount of torque provided to the knee and hip extension depends on the “assist-level” (1–5).

### Participants

In total, 11 able-bodied volunteers without previous experience with wearable robots participated in this study (Table 1). Volunteers with recent lower limb injuries (within 6 months) were excluded. All sessions were conducted by a “Myosuit certified” physiotherapist under the supervision of a licensed medical doctor. All participants provided a written informed consent and research ethics of human experiments was ensured by conducting the sessions in accordance with the contents approved by the Institutional Review Board of Korea University College of Medicine (IRB No. 2021-0120-01). All experiments were carried out in accordance with the approved guidelines and with the Declaration of Helsinki. The raw data supporting the findings of this study are available upon request to the corresponding author. All participants consented to publish the information/image(s) in an online open-access publication.

**Table 1:** Participant information.

Subject	Weight (kg)	Height (cm)	Age	Sex
#1	68	177	26	M
#2	61	158	24	F
#3	50	175	20	M
#4	80	180	29	M
#5	78	178	28	M
#6	86	173	27	M
#7	53	164	24	F
#8	70	177	26	M
#9	45	155	25	F
#10	38	164	28	F
#11	85	175	37	M

## Experimental protocol

In order to assess the biomechanical effects of Myosuit during stair ascent we compared the temporal parameters, plantar force distribution, joint angles, and EMG activation patterns of five stair ascent conditions: no-Myosuit (baseline), Transparency mode, Assist mode assist-level 1, 3, and 5. For each condition, the participants were asked to ascend a flight of stairs (8-treads, depth 25.5 cm × height 17 cm) which was repeated four times. Prior to any data acquisition, the subjects were given a familiarization period allowing them to get accustomed to the different Myosuit settings by walking around on a level surface and ascending stairs. Once, the subjects felt comfortable and ready to proceed with the experiment, they were instructed at the bottom of the stairs to “ascend at a pace which feels comfortable and safe until the top of the stairs”. Data from the first and last step was not included for signal processing in order to discard any initiation and termination strategies, thus, a total of 12 cycles (4 repetitions of 3-cycles) were analyzed for each Myosuit condition.

### Signal processing

The plantar force distribution, lower-limb joint angles, and muscle activation patterns during stair ascent were analyzed by using Pedar-X (Novel GmbH, Munich, Germany), IMU, and EMG sensors (Delsys Inc., Boston, MA, USA), respectively. As it is shown in Fig. 2, the biomechanics of stair ascent can be divided into the following five distinct phases: WA, PU, CN, FC, and FP^12,51–55^. Each phase serves a specific function for successful and continuous negotiation of stairs. Thus, the biomechanical patterns were analyzed in accordance with the respective phases such that Myosuit’s assistive performance and profile could be identified.

**Figure 3:**
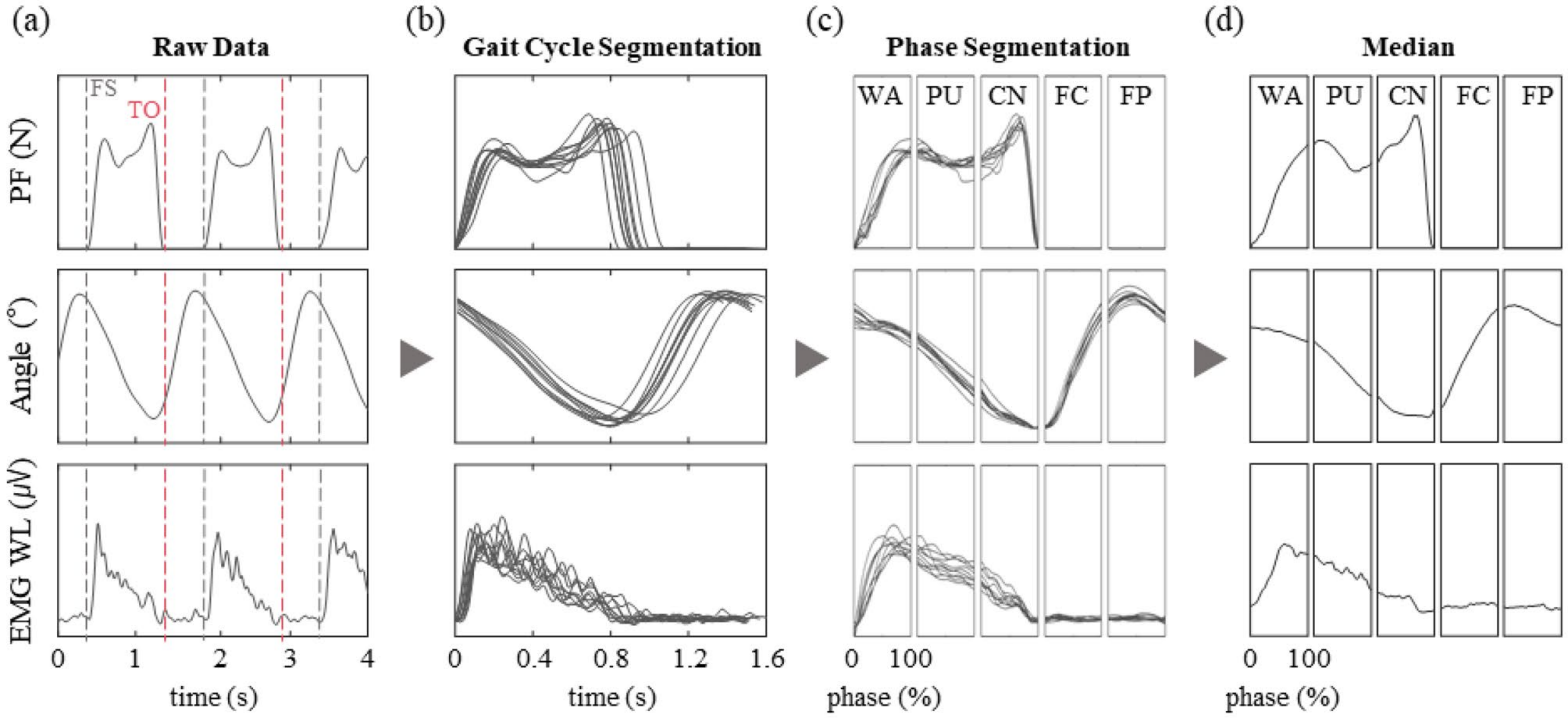
Signal processing scheme. (**a**) The data are first synchronized, and ipsi- and contra-lateral foot-strike (black dash line) and toe-off positions (red dash line) are identified using a simple threshold on the plantar force signal. (**b**) Using the foot strike positions we divide the data divided into individual steps. (**c**) The 5 gait phases of stair ascent are identified, and the data are normalized against time such that each phase can be represented in percentages of phase. (**d**) The median (line) are calculated in order to present a trace that represents a specific gait condition.

The plantar force, IMU, and EMG signals were first synchronized and then a simple threshold on the plantar force was applied to identify the ipsi- and contra-lateral foot-strike and toe-off positions (Fig. 3a, b). The stair ascent cycle starts with the ipsilateral foot-strike and was initially divided into the stance and swing phases, which is separated by the ipsilateral toe-off. The stance phase is further divided into WA, PU, and CN phases by contralateral toe-off and foot-strike, respectively. And the swing phase is equally divided between the FC and FP phase^55^. The ensemble data (Fig. 3c) were then represented as medians such that the gait patterns from different conditions could be overlayed and compared (Fig. 3d).

**Figure 4:**
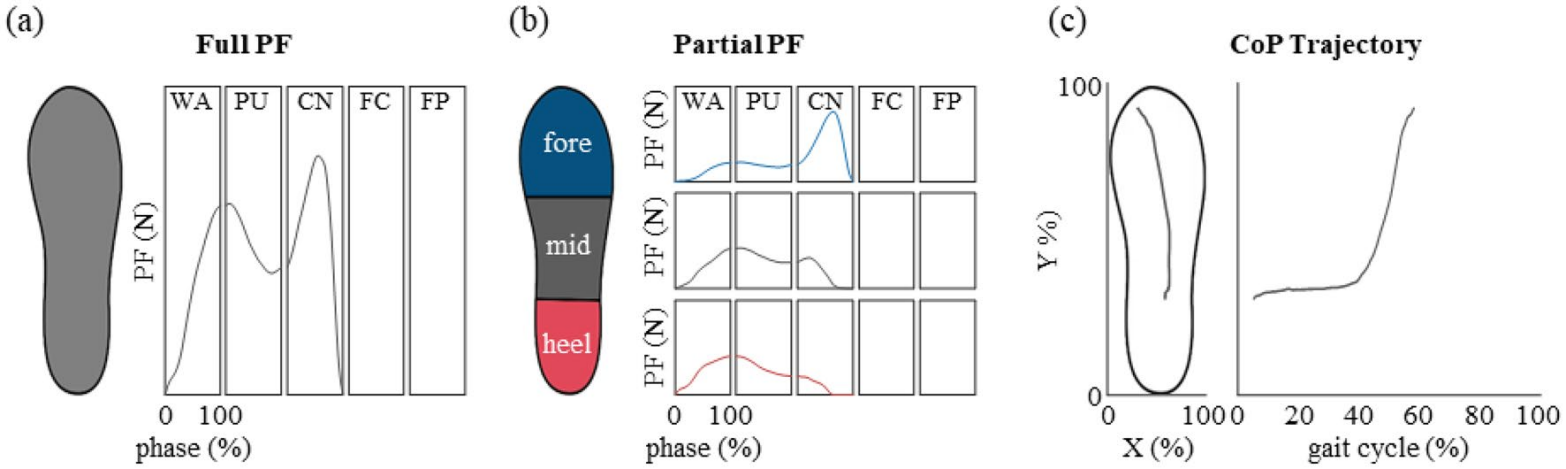
Plantar force analysis. (**a**) The full plantar force obtained from Pedar-X, (**b**) normalized anterior–posterior partial pressure, and (**c**) the CoP trajectory are shown respective to the gait cycle.

Plantar force sensitivity has been closely associated with balance and risk of falling^33,56^. Thus, it is imperative that physiological foot pressure patterns are well facilitated during training for stair ascent. We acquired the subject’s plantar force (Fig. 4a) using Pedar-X insoles (50 Hz) which contain a matrix of 99 capacitive type pressure sensors. Furthermore, using masks, the partial plantar force of the hind-, mid-, and fore-foot were also calculated in order to assess if any intra-foot partial pressure changes were elicited in the anterior–posterior direction depending on the different experimental conditions (Fig. 4b). We also evaluated the center of pressure (CoP) trajectory (Fig. 4c, left insert), provided by Pedar-X insoles, which was then projected upon the y-axis (Fig. 4c, right insert).

In order to analyze the muscle activation patterns during stair ascent, the EMG signals were obtained at 1777 Hz which was the highest frequency available via Delsys Trigno Wireless system (Delsys, Inc, Boston, MA, USA) from the soleus (SL), tibialis anterior (TA), biceps femoris (BF), rectus femoris (RF), VM, and gluteus maximus (Gmax) which have all been well reported to be pivotal for stair ascent (Fig. S1)^16,22^. Due to the high variability of EMG signals, we rectified the raw signals in order to perform quantitative comparisons and analyses. We chose to use the waveform length (WL) because it has been previously reported to correlate well with muscle activation patterns due to its usage of both amplitude and frequency information. A 4th order Butterworth (20 to 500 Hz) was first applied to the raw *EMG* signal and then *WL* was calculated via Eq. (1) shown below:

1 $$EMG WL\left(t\right)=\sum_{n=t-N+2}^{t}\left|EMG\left(n\right)-EMG(n-1)\right|,$$

where $EMG$, $N$, and $t$ is the raw EMG signal, window size, and current sample, respectively.

The knee, hip, and ankle joint sagittal angles were estimated as described in our previous work^45,50^, which is based on the method presented by Saito et al.^57^. The relative angle of the estimated 3D orientation data, provided via the IMU sensors (74 Hz) via Delsys Trigno Wireless system, of the bones that make up the joint was used to estimate the joint angles. The 3D orientation itself was provided via the sensors’ internal acceleration, rotation, and earth magnetic field sensors. The sensors were placed as shown in Fig. S1.

### Group analysis

It is important to present the group characteristics that are observed across the participants. Therefore, each individual’s spatio-temporal, plantar force, and EMG data were subjected to group analysis. The group analysis data were presented as median and 25th and 75th percentiles using the raw spatio-temporal data and normalized plantar force and EMG data. The plantar force and CoP_y_ were normalized with the bodyweight and foot length then presented in percentages, respectively. The *EMG WL* was normalized by obtaining the *normEMG WL*_*condition*_ which is the subjects *EMG WL* divided by the maximum *EMG WL* of the baseline condition as shown below:

2 $$normEMG W{L}_{condition}= \frac{ EMG W{L}_{condition}}{\mathrm{max}( EMG W{L}_{Baseline})} \times 100\%$$

Also, the difference in group EMG levels were assessed relative to the baseline as shown below:

3 $$\Delta EMG W{L}_{Rel}= \frac{mean(normEMG W{L}_{condition})-mean(normEMG W{L}_{baseline})}{mean (normEMG W{L}_{baseline})} \times 100\%$$

### Statistical analysis

Once the data was normalized for each subject the median of the group was acquired and the group data were presented as the median, and the 25th and 75th percentiles. The statistical significance was evaluated by Wilcoxon’s signed rank test. A custom MATLAB (Mathworks Inc, Natick, MA, USA) script was used for all the data processing.

## Results

### Temporal parameters and lower limb kinematics

**Figure 5:**
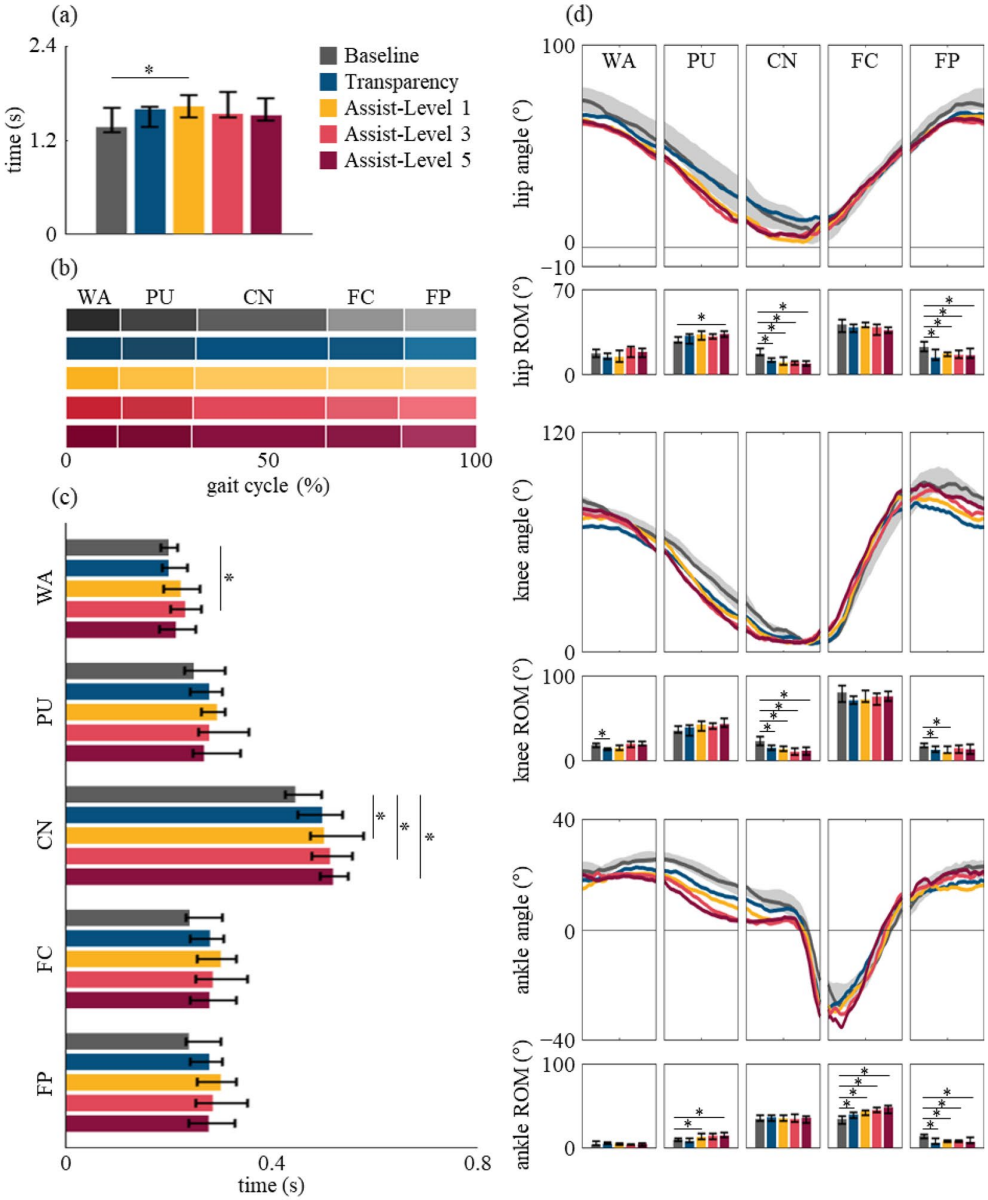
Group analysis of the spatio-temporal parameters. From top to bottom (**a**) the median stride time, (**b**) inter-phase ratio, (**c**) absolute median times for each phase. The data are represented as represented as median (bar) and 25th and 75th percentiles (error bar). (**d**) The estimated hip, knee, and ankle joint angles. The data are represented as represented as median (line) and the shaded areas for the baseline time traces are shown in 25th and 75th percentiles. The data for the bar graphs are represented as represented as median (bar) and 25th and 75th percentiles (error bar). Asterisks represent the significant difference in time or ROM between a specific condition and the baseline assessed by Wilcoxon signed rank test (*p < 0.05).

We first examine the temporal parameters of the participant group during stair ascent while wearing Myosuit in order to assess whether the phases are congruent with that of the baseline condition and if Myosuit is able to facilitate the user’s comfortable gait speed. The median stride time for the baseline is 1.38 s and increases 0.22, 0.26, 0.16, and 0.15 s which translates to approximately 15.94%, 18.84%, 11.59%, and 10.87% for transparent, assist-level 1, 3, and 5, respectively (Fig. 5a). While the overall duration remains relatively consistent, it is important to address whether the inter-phase ratios are also conserved (Fig. 5b, c). The results suggest that no significant delays were elicited for a specific phase but was spread out across the PU, CN, FC, and FP phases (Fig. 5b, c).

The results shown in Fig. 5d also suggest that, qualitatively, no significant differences in lower limb kinematics when ascending stairs with Myosuit is observed. We do note that that the hip reaches maximum extension earlier than the baseline in the CN phase, which is also observable for the knee. It is clearly evident from Fig. 5d that the overall joint kinematics of the different Myosuit conditions well match that of the baseline. Moreover, Myosuit does not interfere with a healthy subject’s comfortable speed even at maximum assist-level 5.

### Plantar force patterns

As we previously mentioned, proper feedback, especially plantar force, is of utmost importance when establishing motor learning and ensuring safety^30,31,33,56^. Because the feet are the only part of the body that interact with the ground during stair ascent and plantar sensitivity is directly associated with risk of falling^33,56^, the plantar force as well as CoP trajectories should be carefully examined.

**Figure 6:**
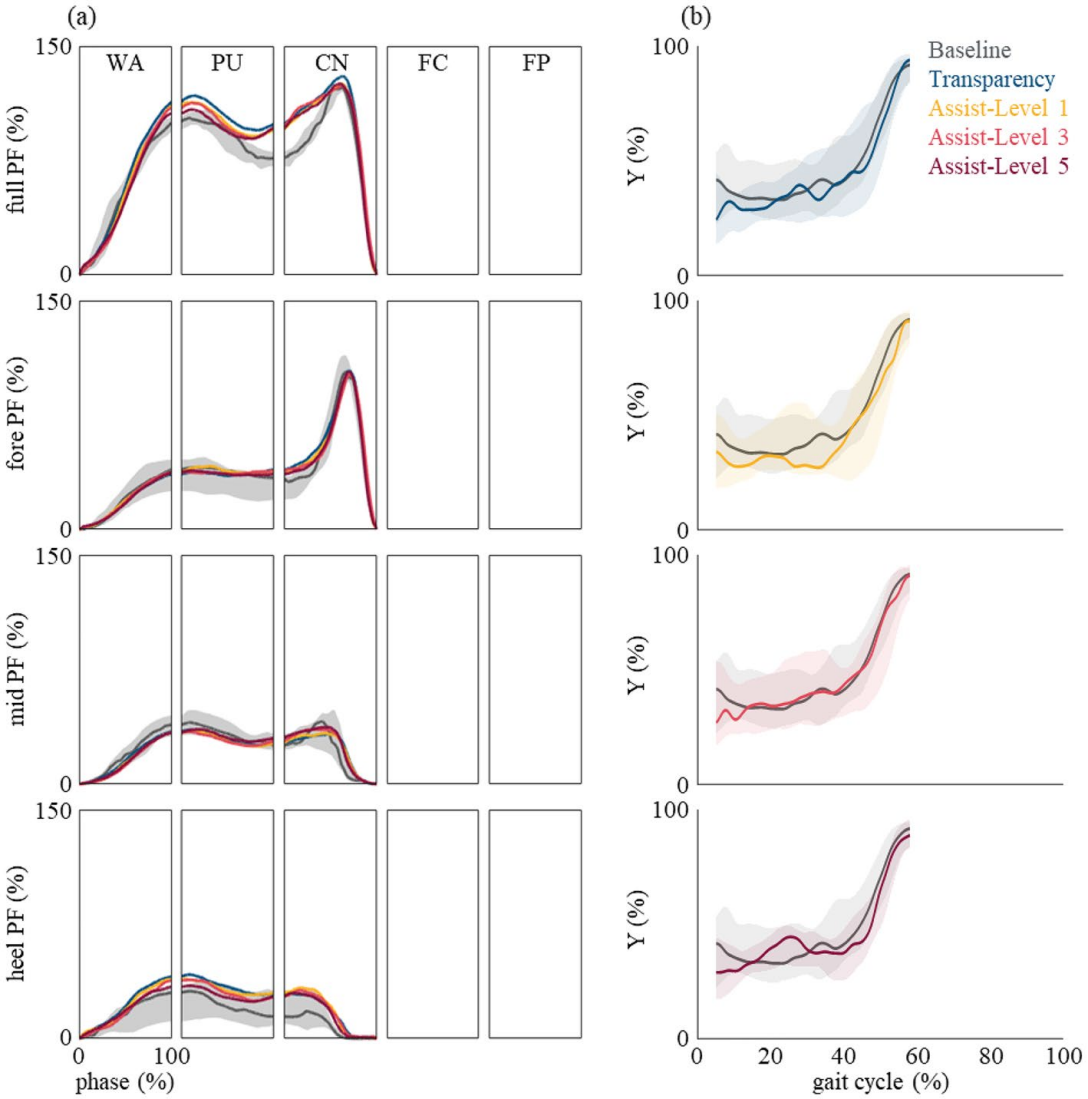
Group analysis of the plantar force patterns. (**a**) From top to bottom total, forefoot, midfoot, and heel plantar force during stair ascent. The data are represented as represented as median (line) and the shaded areas for the baseline time traces are shown in 25th and 75th percentiles. (**b**) The CoP_y_ trajectory during stair ascent. The data are represented as represented as median (line) and 25th and 75th percentiles (shaded area). From top to bottom, Transparency mode, Assist mode assist-level 1, 3, and 5 are shown. The region each the CoP_y_ resides for each condition is similar to that of the baseline.

The plantar force time traces of the group are shown in Fig. 6a. The results suggest that the global plantar force of the various Myosuit conditions remain similar with the baseline. Unlike overground gait which displays clear heel contact and toe off, the initial and terminal instances during stair ascent usually remain within the mid-foot because stair ascent only utilizes the ankle rocker^58^. This provides a larger base throughout the single limb support which in turn provides stability for the PU phase which is when the center of mass (CoM) rises and knee flexion occurs for contralateral limb. Thus, we divided the global plantar force into heel, mid-, and fore-foot regions. Not only the global plantar force but also regional partial plantar force was also similar with the baseline. Furthermore, we evaluated the CoP progressions along the anterior–posterior direction and the results suggest the two time-traces are similar to one another (Fig. 6b)^56^.

### EMG patterns

In order for Myosuit to serve as an RAGT device, it is imperative that a quantitative verification of user assistance be established. In this study, we approach this proposition by evaluating the lower limb *normEMG WL* patterns as well as analyzing the changes elicited in response to Myosuit and the different assist-levels.

**Figure 7:**
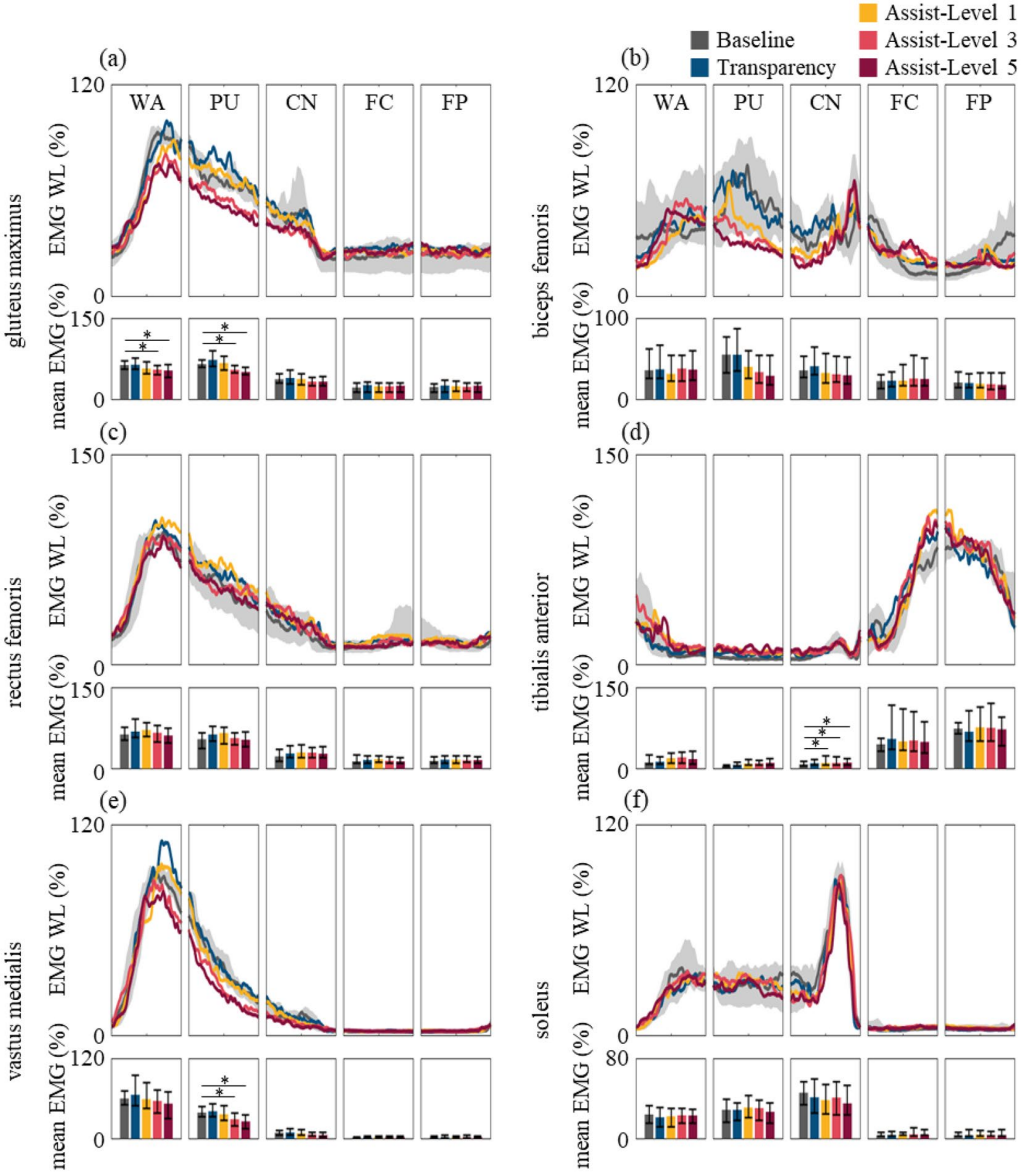
EMG patterns observed during stair ascent performed by subject # 1. The EMG WL time traces and mean EMG WL of the five phases are shown for (**a**) Gmax, (**b**) RF, (**c**) VM, (**d**) BF, (**e**) TA, and (**f**) SL, respectively. The data are represented as represented as median (line) and the shaded areas for the baseline time traces are shown in 25th and 75th percentiles. The data for the bar graphs are represented as represented as median (bar) and 25th and 75th percentiles (error bar). Asterisks represent the significant difference in EMG WL between a specific condition and the baseline assessed by Wilcoxon signed rank test (*p < 0.05).

We find that the overall *normEMG WL* patterns of all evaluated muscles, when wearing Myosuit, were similar to that of the baseline throughout all five phases of stair ascent (Fig. 7). Specifically, we observe dormant hip extensor, knee extensor, and plantar flexor *normEMG WL* at the beginning of the WA phase followed by a coordinated simultaneous increase as body weight is transferred to the ipsilateral limb. Activation of these muscles gradually decay until the end of the CN phase as work against gravity is no longer needed. When the CoM reaches its maximum height and starts to descend, early CN phase, bodyweight is supported by eccentric activation of the plantar flexors^59^. During the swing phase, FC and FP, all evaluated muscles except for TA becomes inactive. This marked increase of the TA is vital for optimal foot clearance as a plantar flexed ankle would require added burden of lifting and placing the foot over the next step^9,30,47^.

**Figure 8:**
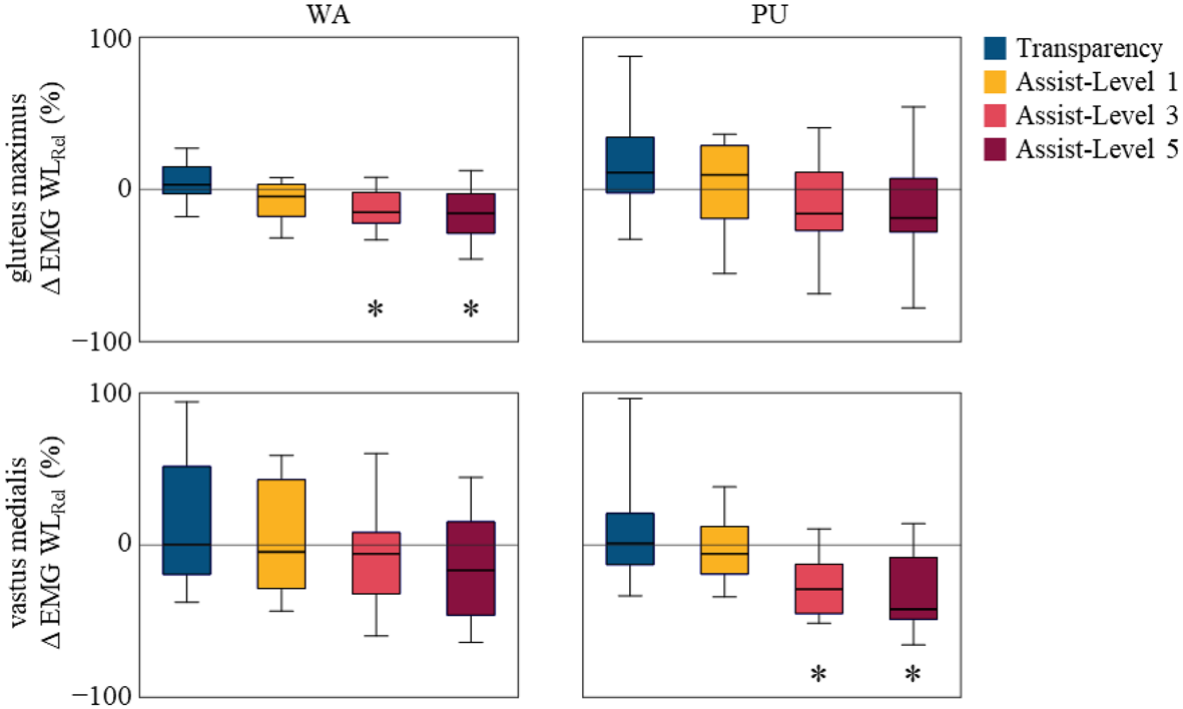
Group analysis of the biomechanical response to the Myosuit. Box plots show ∆*EMG WL*_*Rel*_ for stair ascent conditions of Gmax and VM for WA and PU phase. The data are represented as median (line within box), 25th and 75th percentiles (box), and minimum and maximum values (whiskers). *p < 0.05, assessed using the Wilcoxon signed rank test.

One might ask, if the EMG patterns and subsequent movements are similar, then how is Myosuit assisting the user? In this study we examine whether the lower limb demand was reduced by evaluating the EMG levels. The *∆EMG WL*_*Rel*_, difference in *normEMG WL* of a specific condition (e.g. assist mode level 5 WA phase) normalized against the baseline condition, is −15.57, −12.75, and 2.35% during the WA phase and −21.83, −33.2, and −46.61% during PU phase for Gmax, VF, and BF, for assist-level 5 respectively (Fig. 8 and Table S1). The overall trends observed with both the joint angles and EMG WL time traces were not significantly altered while reducing the demand of raising one’s CoM during stair ascent^60–63^.

## Discussion

In this study, we explored whether Myosuit, a wearable soft robot, could be potentially employed into a stair ascent training regimen by assisting the users with hip and knee extension torque. A total of 12 stair ascent cycles, from four repetitions, for each participant, 11 subjects in total, were analyzed in this experiment. The data analyzed include temporal parameters, plantar force, and CoP_y_ which were acquired via Pedar-X insoles and the kinematics and EMG data which were acquired via trigno wireless systems. As we initially hypothesized, we find that the hip and knee extensor EMG WL values decreased significantly without altering the user’s physiological patterns.

To address our observations in more detail, firstly, it is vital that we point out that the preservation of the user’s natural patterns suggest that Myosuit does not impede with the user’s natural movements. Often, when interventions are introduced, addition of new or exclusion of existing movements occurs^45,47^. When extensive training with these interventions is performed, non-physiological detrimental patterns may be habitualized^64–66^. Secondly, the EMG patterns also well match that of the baseline. This suggests that the Myosuit is causing minimal deviations from the user’s natural intent when ascending stairs. However, in order for Myosuit to serve as an RAGT device, some sort of assistance should be expected. In this study, we find significant reduction in Gmax, BF, RF, and VM, which are hip and knee extensors, EMG levels for assist-level 5. Thirdly, for each phase, the trajectory of the plantar force and CoP_y_ agree well with the baseline which is vital for sensory feedback and safety. Collectively, unaffected temporal patterns, kinematics, sensory feedback, and reduction of knee and hip extensor demand while not disrupting the overall EMG patterns suffice our initial criteria for Myosuit to serve as a stair ascent training robot. Because Myosuit assists hip and knee extension, possible target users could be, but not confined to, the geriatric population and post-surgery patients.

There are some concerns regarding the ~ 5.6 kg weight of Myosuit. It has been previously shown that added weight that exceeds 10% of the body weight significantly increases the task demand^67^. Thus, in our study, any subject that weighs less than 56 kg may feel an added burden that other participants do not experience. While no concrete postulations can be made due to the small sample size, we do observe different patterns of assistance depending on the subject’s weight. If this is the case, the intended assistance profile could be absent based on the subject’s bodyweight, thus, warranting further investigations.

Regarding the hip angle, we would like to point out that our method of estimation has its limitations. We calculated the relative angle between the sternum and femur which would result in erroneous estimations when excessive lumbar flexion or extension is present. Another topic that was not discussed was, the increased peak plantar flexor EMG levels (Fig. 4) which may be burdensome to a non-healthy user. However, considering that muscle mass decrease starts from the proximal region, this may not present itself as a problem to the geriatric user. Thus, collectively, the reduction of either hip or knee extensor demand while maintaining physiological kinematics and plantar force patterns even in the presence of the added mass of Myosuit, at assist-level 5, serves as evidence that assistance was adequately provided. We also note that our experiment was performed on a normal flight of stairs, within a building with everyday traffic, without any ceiling support. It is to our belief that training with Myosuit is in fact truly untethered and allows users to be unrestricted from location.

Albeit the obvious shortcomings, small sample size of healthy subjects, of our study, Myosuit should be ready for real-world application as clinical trials with patients are already being performed^48^. These studies have shown ~ 22% improvements in walking speeds compared to pre- and post- training with Myosuit. However, one must be cautious when assessing the viability of wearable robotics without addressing volitional muscle activation patterns as earlier versions of gait rehabilitation robots did not produce desirable outcomes^68^. Our study contributes by providing unequivocal evidence that the physiological movements as well as motor control intent are facilitated while Myosuit reduces the user’s hip and knee extension demand. It would be natural that the next step is to investigate whether the observations found with healthy subjects translate to the geriatric and post-surgery population.

### Supplementary Information

Supplementary Information.

## Data Availability

The raw data supporting the findings of this study are available upon request to the corresponding author.
