# Supplementary material for: Investigation with able-bodied subjects suggests Myosuit may potentially serve as a stair ascent training robot
Source: Sci Rep. 2023 Aug 29;13:14099. doi: 10.1038/s41598-023-35769-2 (PMC10465530; doi:10.1038/s41598-023-35769-2)
Supplement: Supplementary file 1 — Supplementary Information. [file 41598_2023_35769_MOESM1_ESM.docx]

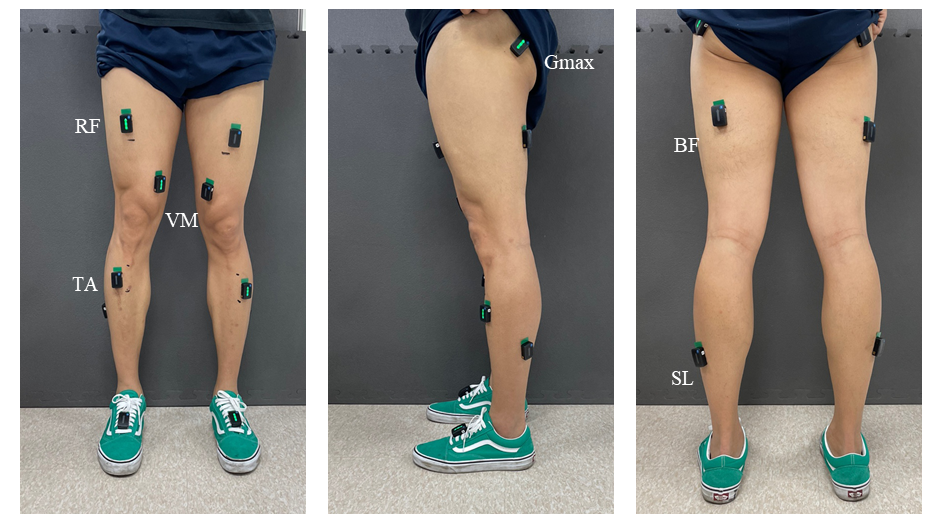


**Figure S1.** The EMG and IMU sensor positions. The sensors were placed on the Gmax, RF, BF, VM, TA, and SL.

**Table S1. Comparison of *normEMG WL***

|  |  | WA | PU | CN | FC | FP |
| --- | --- | --- | --- | --- | --- | --- |
| GMAX | Baseline | 69.09  (60.85, 80.17) | 63.91  (61.51, 68.34) | 41.61  (34.25, 52.11) | 23.53  (13.22, 31.75) | 24.63  (13.8, 29.58) |
|  | Transparency | 70.46  (60.86, 76.74) | 74.16  (63.45, 91.45) | 43.58  (29.54, 64.87) | 27.45  (14.38, 31.82) | 27.76  (14.3, 35.15) |
|  | Assist-Level 1 | 57.43  (44.39, 72.81) | 67.04  (57.07, 83.55) | 42.34  (28.54, 51.33) | 24.51  (14.74, 30.93) | 25.36  (15.28, 34.1) |
|  | Assist-Level 3 | 56.75*  (50.17, 64.78) | 54.54*  (50.63, 60.14) | 34.64  (29.74, 40.62) | 24.6  (14.97, 31.36) | 24.34  (15.05, 30.98) |
|  | Assist-Level 5 | 52.83*  (35.88, 64.3) | 51*  (49.8, 55.36) | 36.21  (30, 41.96) | 25.16  (14.6, 32.11) | 27.19  (14.54, 29.93) |
|  |  | WA | PU | CN | FC | FP |
| RF | Baseline | 65.61  (61.8, 82.66) | 56.72  (39.51, 65.27) | 24.66  (15.12, 38.02) | 14.36  (11.13, 20.79) | 14.75  (10.06, 21.71) |
|  | Transparency | 77.57  (69.41, 89.29) | 65.14  (51.78, 79.54) | 28.51  (23.11, 42.96) | 17.6  (11.63, 23.23) | 17.22  (10.41, 23.72) |
|  | Assist-Level 1 | 80.98  (76.5, 87.58) | 71.33  (49.2, 78.38) | 31.01  (22.17, 46.09) | 20.17  (12.17, 23.53) | 15.92  (11.39, 24.19) |
|  | Assist-Level 3 | 81.09  (58.98, 86.09) | 56.13  (45.34, 66.17) | 29.49  (20.53, 36.76) | 13.75  (10.85, 22.37) | 13.51  (11.08, 23.22) |
|  | Assist-Level 5 | 69.25  (50, 79.62) | 55.18  (38.28, 67.09) | 31.06  (22.52, 42.71) | 13.84  (11.05, 20.37) | 15.53  (11.46, 21.86) |
|  |  | WA | PU | CN | FC | FP |
| VM | Baseline | 72.41  (65.66, 78.24) | 40.14  (30.34, 47.82) | 9.88  (6.54, 13.18) | 2.73  (1.57, 3.85) | 2.5  (1.65, 3.71) |
|  | Transparency | 75.18  (48.64, 98.21) | 36.25  (27.38, 46.93) | 8.78  (6.32, 16.43) | 2.67  (2.09, 4.75) | 2.85  (2.02, 5.09) |
|  | Assist-Level 1 | 65.36  (45.63, 90.55) | 31.75  (25.89, 51.91) | 7.68  (4.1, 15.88) | 2.87  (2.34, 5.06) | 3.08  (2.15, 5.19) |
|  | Assist-Level 3 | 67.45  (40.92, 78.9) | 24.86*  (17.98, 31.99) | 5.97  (4.34, 9.22) | 2.76  (2.29, 5.24) | 2.63  (2.16, 4.62) |
|  | Assist-Level 5 | 58.67  (38.3, 76.11) | 24.52*  (15, 27.6) | 5.58  (3.71, 9.13) | 2.9  (2.1, 4.29) | 3.01  (1.93, 4.43) |
|  |  | WA | PU | CN | FC | FP |
| BF | Baseline | 37.72  (27.37, 56.36) | 69.01  (34.57, 75.42) | 35.81  (25.27, 45.15) | 17.41  (11.47, 26.5) | 18.01  (13.69, 30.75) |
|  | Transparency | 38.4  (26.49, 69.92) | 46.67  (39.43, 87.65) | 39.88  (33.39, 57.93) | 21.45  (16.39, 35.67) | 21.79  (14.01, 30.88) |
|  | Assist-Level 1 | 29.51  (23.68, 55.76) | 40.76  (26.22, 56.98) | 27.72  (18.42, 43.38) | 23.35  (17.84, 38.58) | 17.79  (13.77, 33.09) |
|  | Assist-Level 3 | 43.78  (27.34, 58.43) | 32.25  (18.98, 48.62) | 24.01  (20.01, 39.22) | 24.66  (15.81, 50.38) | 17.56  (12.82, 31.93) |
|  | Assist-Level 5 | 43.66  (23.75, 62.6) | 28.04  (17.48, 55.63) | 19.64  (16.73, 39.2) | 25.05  (16.57, 47.87) | 17.93  (14.4, 33.53) |
|  |  | WA | PU | CN | FC | FP |
| TA | Baseline | 9.21  (7.19, 14.75) | 5.11  (4.22, 7.01) | 8  (6.95, 9.33) | 44.84  (25.8, 58.41) | 79.84  (72.14, 82.34) |
|  | Transparency | 10.91  (9.95, 16.16) | 7.05  (6.33, 12.14) | 9.03  (6.48, 12.7) | 54.1  (31.97, 105.57) | 68.18  (57.04, 103.1) |
|  | Assist-Level 1 | 17.15  (10.48, 26.86) | 9.88*  (7.31, 16.54) | 10.74  (7.46, 22.15) | 40.66  (27.14, 104.59) | 81.78  (54.58, 107.53) |
|  | Assist-Level 3 | 17.26  (9.43, 32.71) | 9.97*  (7.52, 15.05) | 10.87  (8.56, 19.28) | 58.52  (27.44, 97) | 79.56  (54.07, 122.86) |
|  | Assist-Level 5 | 14.39  (8.19, 28.18) | 13.89*  (7.15, 16.89) | 11.68  (8.11, 15.89) | 46.1  (18.06, 89.59) | 83.01  (42.24, 101.42) |
|  |  | WA | PU | CN | FC | FP |
| SL | Baseline | 27.63  (16.97, 38.34) | 28.57  (17.23, 39.46) | 39.85  (32.41, 51.84) | 3.93  (2.9, 5.86) | 4.08  (2.95, 6.52) |
|  | Transparency | 24.96  (11.01, 33.41) | 30.93  (18.09, 34.21) | 34.25  (18.5, 40.22) | 4.13  (3.33, 6.89) | 3.6  (2.92, 9.27) |
|  | Assist-Level 1 | 25.88  (8.33, 33.55) | 31.3  (20.08, 42.07) | 31.56  (18.4, 34.7) | 4.49  (3.74, 6.39) | 4.76  (2.94, 8.49) |
|  | Assist-Level 3 | 26.17  (17.22, 31.98) | 32.23  (19.93, 39.8) | 24.24  (19.02, 36.4) | 4.71  (3.55, 6.65) | 4.23  (3.08, 7.66) |
|  | Assist-Level 5 | 25.56  (15.14, 29.81) | 33.36  (16.54, 35.7) | 27.26  (19.03, 36.21) | 4.54  (3.84, 9.22) | 3.99  (3.14, 8.97) |

Asterisks represent the significant difference in *EMG* *WL* between a specific condition and the baseline normalized against the baseline in percentages. Statistical significance was assessed by Wilcoxon signed rank test (**p* < 0.05).


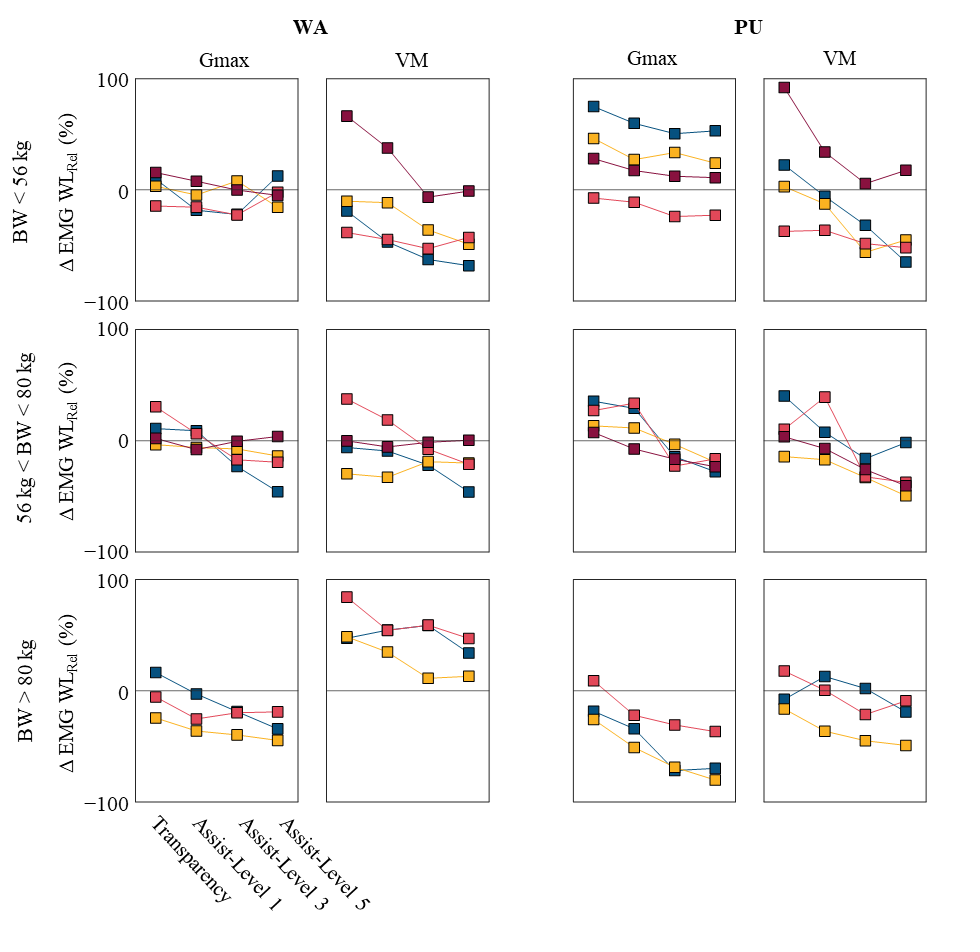


**Figure S2.** Weight dependent assistive profile. *∆EMG WL_Rel_* for the WA (left two columns) and PU (right two columns) phases. The 4 subjects that weigh less than 56 kg display assistance for knee flexors while added burden is observed for Gmax. The opposite is observed for the 3 subjects that weigh more than 80 kg. The data represent the median of *∆EMG WL_Rel_*.
